# Prognostic Factors and Survival in Patients Treated Surgically for Recurrent Metastatic Uterine Leiomyosarcoma

**DOI:** 10.1155/2014/919323

**Published:** 2014-06-22

**Authors:** Han L. T. Hoang, Kelsey Ensor, Gerald Rosen, H. Leon Pachter, Joseph S. Raccuia

**Affiliations:** ^1^Department of Surgery, NYU Langone Medical Center, 560 First Avenue, Suite 6C, New York, NY 10016, USA; ^2^NYU School of Medicine, 550 First Avenue, New York, NY 10016, USA; ^3^Department of Medicine, NYU Langone Medical Center, 240 East 38th Street, FI 19, New York, NY 10016, USA

## Abstract

*Background*. Uterine leiomyosarcoma (LMS) is a rare diagnosis, which is seldom cured when it recurs with metastatic disease. We evaluated patients who present with first time recurrence treated surgically to determine prognostic factors associated with long-term survival. *Methods*. Over a 16-year period, 41 patients were operated on for recurrent uterine sarcoma. Data examined included patient age, date of initial diagnosis, tumor histology, grade at the initial diagnosis, cytopathology changes in tumor activity from the initial diagnosis, residual tumor after all operations, use of adjuvant therapy, dates and sites of all recurrences, and disease status at last followup. *Results*. 24 patients were operated for first recurrence of metastatic uterine LMS. Complete tumor resection with histologic negative margins was achieved in 16 (67%) patients. Overall survival was significantly affected by the FIGO stage at the time of the initial diagnosis, the ability to obtain complete tumor resection at the time of surgery for first time recurrent disease, single tumor recurrence, and recurrence greater than 12 months from the time of the initial diagnosis. Median disease-free survival was 14 months and overall survival was 27 months. *Conclusion*. Our findings suggest that stage 1 at the time of initial diagnosis, recurrence greater than 12 months, isolated tumor recurrence, and the ability to remove ability to perform complete tumor resection at the time of the first recurrence can afford improved survival in selected patientsat the time of the first recurrence can afford improved survival in selected patients.

## 1. Introduction

Uterine leiomyosarcoma (LMS) is a rare diagnosis and the majority of cases are diagnosed after surgery for uterine fibroid disease. These patients are often observed for long periods of time, and not infrequently percutaneous embolization is utilized in an effort to avoid surgery for large symptomatic fibroid disease. Hysterectomy is usually performed only when the fibroids become excessively symptomatic with pain or bleeding [1]. More recently the potential negative effects of uterine morcellation with fibroid surgery have been described in consideration of this rare entity [2]. The incidence of uterine LMS is unusual and represents about 1% of all uterine malignancies [3, 4], and the vast majority of cases are generally confined to the uterus and cervix at the time of diagnosis [5–7].

Uterine LMS must be distinguished from other mesenchymal tumors, such as endometrial stromal sarcoma, adenosarcoma, carcinosarcoma, epithelial tumors, and dedifferentiated mixed tumors as the biology, patterns of recurrence, overall behaviors, and response to treatment are distinct from each other [8–10]. Hence, a separate FIGO classification identifies uterine LMS as entity from the other types of uterine malignancies (Table 1). And although the differences in staging between uterine LMS and endometrial stromal sarcoma are subtle, the staging classification between these two diseases is distinct [11]. In this report we examine only those cases with histologically documented recurrent uterine LMS in an attempt to establish prognostic factors for survival in these patients.

Uterine LMS is an aggressive disease and survival after initial diagnosis has been reported to be 50% in stage 1 and stage 2 disease and dismal in advanced-stage disease [12–15]. The most important prognostic factor is stage, followed by age, mitotic count, and tumor size [8, 16]. Adjuvant therapies such as chemotherapy and radiation seem to have little effect on overall survival [8, 17–19]. The role of radiation therapy is extremely limited as it has only been examined in the adjuvant setting after the initial diagnosis and has been associated with decreased local recurrence but has no effect on overall survival [8, 17–19]. Chemotherapy, such as dacarbazine, gemcitabine, and docetaxel, has been associated with slightly improved survival [18, 20]; however, the role of adjuvant medical therapy with recurrent disease is almost negligible.

In most patients with extrauterine disease, recurrence occurs within 6 to 18 months from the initial diagnosis [4, 21, 22] and surgery seems to be the only treatment that has been demonstrated to improve survival in patients with resectable recurrent disease [21]. Despite the limited data in this regard [9, 22, 23] survival of patients with recurrent disease is poor; however, secondary cytoreductive surgery did show benefits in selected patients with pulmonary, abdominal, and pelvic surgery [21–23]. Median survival of patients after secondary cytoreductive surgery was 71% at 2 years [6] and a median survival of 2 years versus 1.1 years in comparison to those only treated medically [22]. Prolonged disease-free interval and complete tumor resection were associated with longer survival [22, 23].

Herein, we described a series of first time recurrences treated primarily with surgery. The overall survival is examined in order to ascertain possible factors that contribute to improved outcomes.

## 2. Methods

All patients who underwent resection for the first recurrence of uterine LMS were reviewed from an ongoing retrospective database over a 16-year period from 1997 to 2013 and approved by the Institutional Review Board at our institution. Comprehensive data was collected, including age, date of initial diagnosis, tumor histology and grade, stage, size, adjuvant therapies, and followup disease status from the initial diagnosis as well after surgery for recurrent disease. The pathology slides from the initial diagnosis were reexamined and the diagnosis of uterine LMS was verified, while definitively excluding epithelial tumors, carcinosarcomas, endometrial stromal tumors, adenosarcomas, and undifferentiated uterine sarcomas. Slides from the initial surgery were also compared to the specimens from the recurrent surgery and examined for histologic changes in cellular atypia and more aggressive patterns in appearance and alterations in tumor heterogeneity.

Staging at the time of the initial diagnosis was applied using the revised international federation of gynecology and obstetrics (FIGO) staging system specifically for uterine LMS [11]. Patients before 2009 were retrospectively restaged using the newer system and then prospectively thereafter. The time of first recurrence was calculated from the time of the initial surgery to the date of the surgical resection of the recurrence. Preoperative imaging was evaluated and only patients who were considered to have resectable disease underwent surgery. Postresection survival was determined from the time of the first recurrence to the last followup. Complete resection without evidence of macroscopic disease and microscopically negative tumor margins on final pathology was defined as a complete resection. Residual disease was defined as microscopic disease on final pathology and incomplete tumor removal was defined as known macroscopic disease not removed at the time of surgery for the first recurrence.

A total of 9 variables were analyzed for possible prognostic value. These included extent of surgical resection, initial tumor stage by FIGO, time to first recurrence, tumor grade, type of procedure, site of first recurrence, single versus multiple recurrences, local versus distant recurrence, postoperative adjuvant therapy after initial diagnosis (none versus chemotherapy and/or radiation), and adjuvant therapy after recurrence.

Survival curves were estimated using Kaplan-Meier method and *P* values were generated using the log rank (Mantel-Cox) ratio as described by Mantel [24]. All statistical analyses were performed using SPSS software (SPSS, version 20, IBM, Chicago, Illinois, USA).

## 3. Results

A total of 24 patients were operated on for recurrent metastatic uterine LMS with a median age of 57 years (37 to 82 years). Single tumor recurrence occurred in 12 (50%) patients while the others (50%) had 2 or more lesions. Overall survival was 2.6 years and survival at 2, 5, and 10 years was 57%, 24%, and 12%, respectively.

Surgery included abdominal, pelvic, and thoracic surgery either alone or in sequence if the metastases were considered resectable. A total of 4 (15%) patients had thoracic procedure alone. Surgery alone, either abdominal or thoracic, was performed in 10 (42%) patients, while 14 (58%) others had a combination of surgery plus chemotherapy (*n* = 13) and the addition of radiation preoperatively in 1 (4%) other. Complete resection, with microscopically confirmed negative margins on final pathology, was achieved in 16 (67%) patients.

Of those with isolated recurrences 4 (16%) had isolated lung resections, 4 (16%) had intestinal resections, 2 (8%) had partial hepatectomies, 1 (4%) had distal pancreatectomy, and 1 (4%) had complete psoas muscle resection. In patients that had multiple site recurrences 5 (20%) had multiple lesions resected from the pelvis (urinary bladder, rectum, and colon), 4 (16%) had multiple intra-abdominal intestinal resections, 1 (4%) had colon and liver resection, 1 (4%) had colon and lung resection, 1 (4%) had bladder and lung resection, and 1 (4%) other had liver and lung resection. There was one (4%) operative death and 6 (25%) other nonfatal complications. Of the nonfatal complications 3 (12%) had prolonged small bowel ileus, 1 (4%) with a small bowel fistula that resolved with expectant management, 1 (4%) with sepsis, and 1 (4%) who had a permanent foot drop from peroneal nerve resection for recurrence involving the psoas muscle.

There were 4 (17%) patients with low-grade tumors and 20 (83%) which were high grade. The grade at the time of the initial diagnosis did not have a significant impact on survival. However, 5 (21%) patients had documented histologic change in the aggressiveness of the cytologic characteristics and cellular atypia at the time of recurrence, which was significantly different when compared to the histologic slides from the initial diagnosis. Three of the four patients with initial low-grade tumors became high grade, and 2 other patients with initial high-grade tumors had significantly more aggressive appearance on cytologic examination after surgery for the recurrence. These more aggressive forms of leiomyosarcoma were also strongly associated with a trend towards decreased survival; however, in comparison to those that had no change in cytopathologic features, the difference did not reach statistical significance. The clinical characteristics of the patients at the time of the initial diagnosis and at the time of the first recurrence are listed (Table 2).

The median time to first recurrence was 26 months (range: 1 to 184 months) from the initial diagnosis of LMS, and the median followup after surgical resection for first time recurrence was 34 months (range: 2 to 89 months).

**Figure 1:**
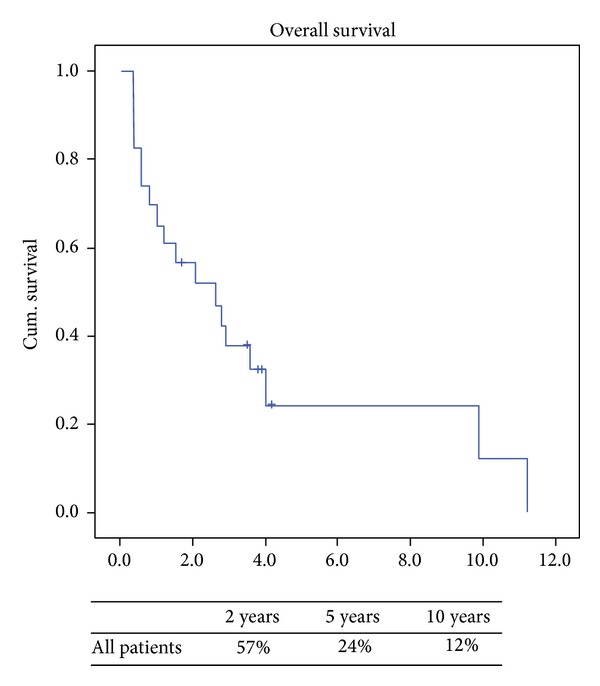


**Figure 2:**
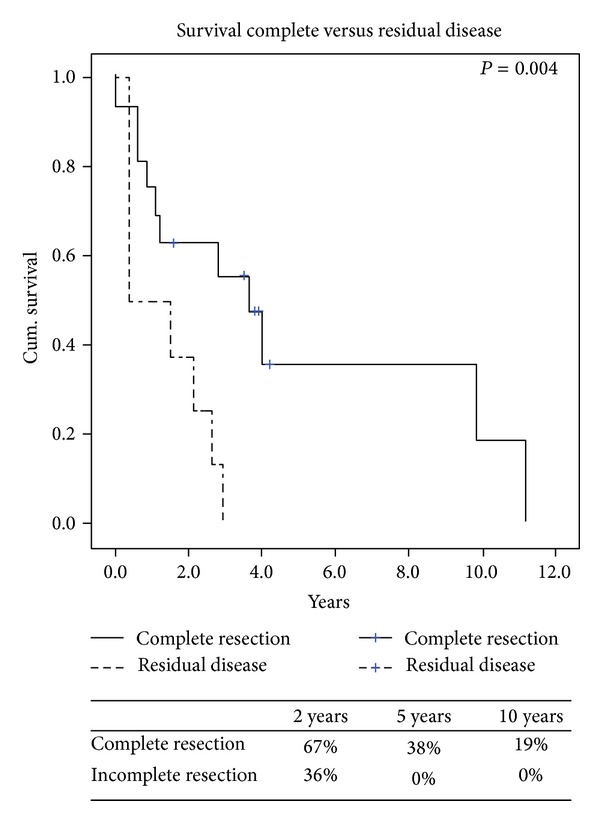


**Figure 3:**
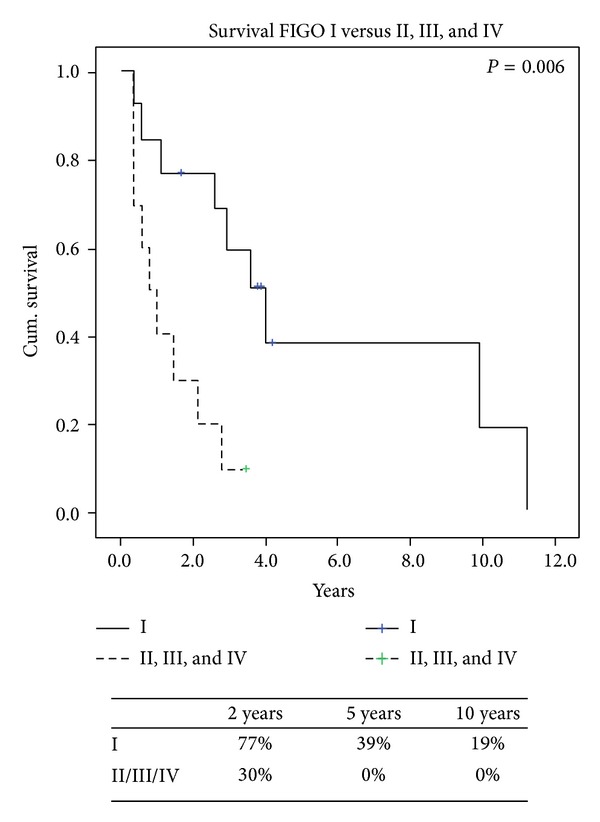


**Figure 4:**
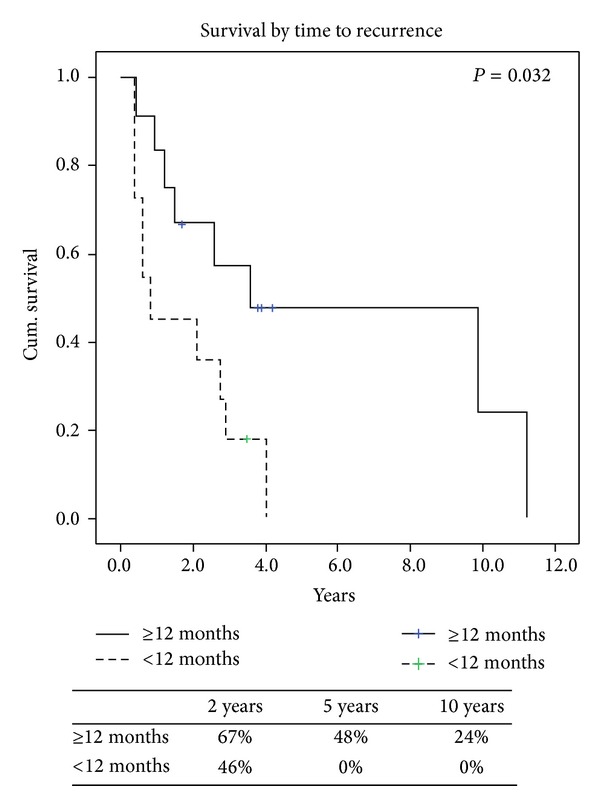


**Figure 5:**
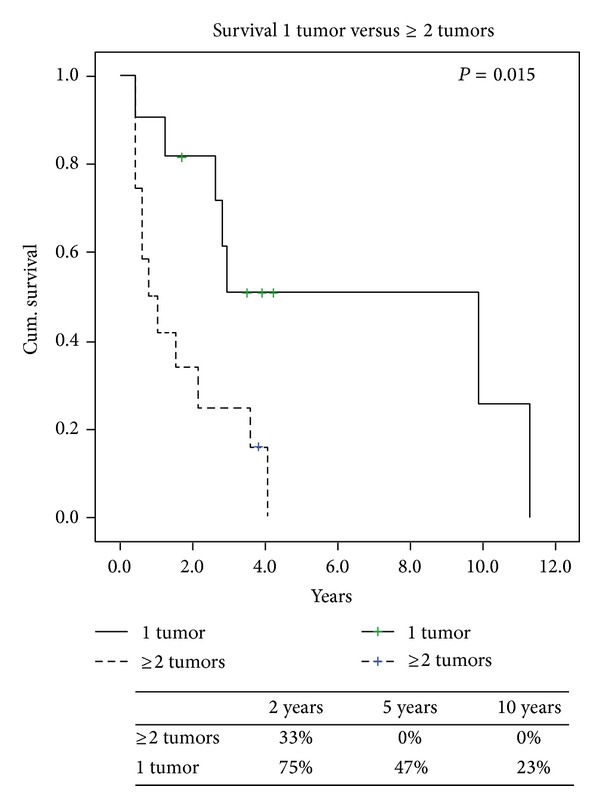


Overall survival from the time of recurrence is presented (Figure 1) with the specific variables listed separately (Table 3). Comparing local versus distant recurrences resulted in no significant difference in survival (*P* = 0.6). Patients with isolated lung metastases after thoracic surgery did not have survival statistically different from the others (*P* = 0.89). However, a negative histologic margin in those with documented complete resections in 16 (67%) patients had significantly better survival when compared to those who had microscopic and macroscopic residual disease (Figure 2). At last followup a total of 5 (21%) patients were alive, of which 3 (13%) were alive without disease. Also, FIGO staging of uterine LMS at initial time of diagnosis did have a predictive impact on overall survival. When we compared FIGO stage I to FIGO stages II, III, and IV those with earlier stage I disease had significant increased 2-year survival of 77% versus 30% (*P* = 0.01) (Figure 3). Time to first recurrence greater than 12 months (Figure 4) significantly affected survival with a median survival of 25 versus 3 months for those diagnosed with a greater time interval between the initial diagnosis and the first recurrence (*P* = 0.006). It is worth noting that all patients that recurred within 12 months had high-grade tumors at initial diagnosis. Single tumor recurrence significantly affected survival (Figure 5) with a median survival of 119 months for single lesions versus 10 months for those with 2 or more tumors (*P* = 0.015). Overall median disease-free survival was 14 months and overall median survival was 27 months. At last followup, 2 patients were alive without evidence of disease, 3 were alive with evidence of disease, and the 19 others died from their disease.

Of the long-term survivors there were 3 (13%) patients that had recurrence more than 5 years after the initial diagnosis and these patients demonstrated the longest survival of the whole group. Patient 1 recurred 15 years after her initial diagnosis of a low-grade uterine leiomyosarcoma with an isolated lung lesion consistent with her primary cancer. Patient 2 with low-grade disease had her first recurrence 6 years after initial diagnosis. She ultimately experienced multiple localized recurrences, which were treated with subsequent surgeries; however, she died of disease 16 years after her initial diagnosis. Patient 3 with initially diagnosed high-grade tumor had a first recurrence 6 years later involving the distal pancreas and another lesion on the soft tissue of the posterior chest wall. These were resected with distal pancreatectomy and chest wall excision. She later recurred with an isolated tumor in the remnant head of the remnant pancreas but refused further treatment. She remains alive with disease 10 years after her initial diagnosis. In all three of these long-term survivors the tumor grade did not change at the time of the first recurrence.

The type of procedure performed, the site of first recurrence, and the use of adjuvant therapy either at the time of the initial diagnosis or after the recurrence had no significant impact on survival (Table 2). Multivariate analysis was not performed due to the small numbers of cases. Perioperative factors impacting survival were examined (Table 3).

The overall survival was significantly affected by the FIGO stage at the time of the initial diagnosis, the ability to obtain complete resection with histologic normal margins at the time of surgery for the recurrent disease, recurrence greater than 12 months from the time of the initial diagnosis, and single tumor recurrence.

## 4. Discussion

Uterine LMS affects 6 out of 1 million women annually [3–5] and, although rare, this is a deadly disease with a recurrence rate often greater than 60% [5, 6, 13, 23]. The series examining similar groups are small; however, surgery seems to be the treatment of choice for those with first time recurrent disease in selected patients. Specifically, those with resectable disease from initial low stage disease and low-grade tumors might benefit the most with operative cytoreduction in this setting [4, 6, 7, 13]. Due to the limited number of patients affected and known benefits of surgical treatment, randomized controlled studies are not feasible. Hence, management and prognostic factors have only been determined based on retrospective data [4, 7, 13, 14]. Furthermore, uterine LMS is a separate biologic entity with a different prognosis from the other uterine sarcomas and it must be uniquely separated from other primary uterine sarcomas. Establishing a histologic confirmation of this diagnosis is paramount for initiating the proper therapy.

The rarity of this disease with the small sample size and paucity of published data concerning first time recurrence for uterine leiomyosarcoma limits extensive evaluation. Studies exclusively limiting evaluation of patients with histologically confirmed uterine LMS where diagnoses such as endometrial stromal sarcoma, adenosarcoma, and carcinosarcoma were excluded from analysis are rare. This is an extremely important variable when analyzing other reports in comparison since these other above entities, although being sarcomas, have completely different biologic behaviors and responses to medical and surgical therapies. Confirmation of those with uterine LMS as a separate subset is invaluable in establishing prognostic variables for this disease and cannot be overemphasized. Due to the paucity of published data, prognostic factors as a guide for management options are limited [18, 22, 23, 25, 26]. Once recurrence has been diagnosed, the reported survival of these patients is as low as 5 months [13]. Only a few have reported outcome and prognostic factors of patients with first time recurrence solely for uterine LMS [22, 23].

Outlined are the comparisons between our report and the other similar publications [22, 23] evaluating first recurrence for uterine LMS (Table 4). The variables with patient demographics and treatment are similar to some minor variations. For example, the time to first recurrence was longer in this report; however, the use of perioperative medical therapy was generally similar. Our rate of complete resection was lower (67%) than the others reported. This could be related to the fact that in our study we had notably fewer patients with single tumors (46%) and multiple site recurrences (54%). However, the outcomes with overall survival were similar. Significant variables associated with prolonged survival included the ability to completely resect the recurrent disease, greater length of time (>12 months) to the first recurrence, and the FIGO stage at the time of the initial diagnosis. Conversely, factors that did not affect survival in our study were the grade of the tumor, type of procedure performed, local versus distant recurrence, and the use of chemotherapy and/or radiation in the perioperative period. Leitao et al. [22] reported similar variables that did not affect survival and they included tumor grade, pelvic versus extrapelvic recurrence, thoracic versus nonthoracic surgery, or the use of chemotherapy and/or radiation at the time of the initial diagnosis or in the perioperative period at the time of the first recurrence. The overall survival at 2 and 5 years in the report by Giuntoli et al. [3] was consistent without data. Leitao et al. [22] reported a higher survival at 2 years of 70%.

Overall survival at 2 and 5 years in the report by Giuntoli et al. [23] was consistent with our data; however, the report by Leitao et al. [22] had increased survival at 2 years of 70%. Survival at 5 years was similar between both the studies and our results. When we examine the survival at 10 years Giuntoli et al. report an overall survival of 20% while ours was 12%. Our survival at 10 years was lower and could be attributed to more recurrences at multiple sites in our study, which could explain the lower number of patients having complete resection. We did have a longer time span to first recurrence (26 months) versus 15 and 16 months, respectively, for the reports [22, 23].

In conclusion, uterine LMS is a rare disease with extremely poor prognosis. For selected patients who had time to first recurrence greater than 12 months, resectable disease at recurrence, and early FIGO stage (stage 1) at the initial diagnosis, secondary cytoreductive surgery can be beneficial and may prolong survival in selected.

## Figures and Tables

**Table 1:** Staging for uterine sarcoma (leiomyosarcomas, endometrial stromal sarcomas, adenosarcomas, and carcinosarcomas).

Stage	Definition
(1) Leiomyosarcoma	
I	Tumor limited to uterus
IA	<5 cm
IB	>5 cm
II	Tumor extends to the pelvis
IIA	Adnexal involvement
IIB	Tumor extends to the extrauterine pelvic tissue
III	Tumor invades abdominal tissue (not just protruding into the abdomen)
IIIA	One site
IIIB	>one site
IIIC	Metastases to pelvic and/or para-aortic lymph nodes
IV	
IVA	Tumor invades bladder and/or rectum
IVB	Distant metastases

(2) Endometrial stromal sarcomas (ESS) and adenosarcomas∗	
I	
IA	Tumor limited to uterus
IB	Tumor limited to the endometrium/endocervix no myometrial invasion
IC	More than half myometrial invasion
II	Tumor extends to the pelvis
IIA	Adnexal involvement
IIB	Tumor extends to the extrauterine pelvic tissue
	Tumor extends to the extrauterine pelvic tissue
III	Tumor invades abdominal tissue (not just protruding into the abdomen)
IIIA	One site
IIIB	>one site
IIIC	Metastases to pelvic and/or para-aortic lymph nodes
IV	
IVA	Tumor invades bladder and/or rectum
IVB	Distant metastases

(3) Carcinosarcomas	
Carcinomas should be staged as carcinomas of the endometrium	

*Note: simultaneous tumors of the uterine corpus and ovary/pelvis in association with ovarian/pelvic endometriosis should be classified as independent primary tumors (from  [11]).

**Table 2:** 

Characteristics	Number	%
57 years (37–82)		

FIGO stage		
I	14	58%
II	4	17%
III	3	13%
IV	3	13%
Grade		
Low	4	17%
High	20	83%
Treatment at initial diagnosis		
Surgery alone	12	50%
Surgery + (CT and/or RT)	12	50%
Time to first recurrence after initial diagnosis		
<12 months	13	54%
≥12 months	11	46%
Single isolated recurrence		
Cervix	3	13%
Lung	4	17%
Intestines	4	17%

	11	46%

Multiple site recurrence		
Abdomen∗/lung/bone	2	8%
Pelvis/lung	2	8%
Pelvis/intestines	3	13%
Retroperitoneum	6	25%

	13	54%

	24	100%

1 tumor versus ≥2 tumors		
1 tumor	12	50%
≥2 tumors	12	50%
Treatment at first recurrence		
Surgery alone	10	42%
Surgery + (CT and/or rads)	14	58%
Resection		
Complete resection	16	67%
Residual microscopic	3	13%
Residual macroscopic	5	21%

*One with bone metastasis.

**Table 3:** 

Survival variables	Number	2 years	5 years	10 years	*P* value
Overall	24	33%	27%	9%	

Time to fist recurrence (months)					
≥12 months	11	77%	39%	19%	0.0060
<12 months	13	30%	0%	0%	
Extent of surgical resection					
Complete	16	53%	42%	16%	0.004
R1/R2	8	12%	0%	0%	
FIGO stage at diagnosis					
I	14	47%	47%	16%	0.010
II/III/IV	10	12%	0%	0%	
1 tumor versus ≥2 tumors					
1 tumor	12	81%	51%	26%	0.015
≥2 tumors	12	33%	0%	0%	
Grade					
Low	4	50%	50%	25%	0.140
High	21	29%	22%	11%	
Type of procedure					
Thoracic only	20	75%	75%	75%	0.890
Nonthoracic	4	26%	0%	0%	
Local versus distant recurrence					
Local	8	63%	25%	0%	0.600
Distant	16	56%	25%	13%	
Adjuvant therapy after initial Dx					
None	12	58%	20%	20%	0.425
CT and/or RT	12	58%	25%	0%	
Adjuvant therapy after recurrence					
None	10	50%	36%	18%	0.565
CT and/or RT	14	70%	13%	13%	

**Table 4:** 

Author, year	Leitao et al., 2002 [22]	Giuntoli et al., 2007 [23]	This study
Patients			
Number	41	80	24
Time to 1st recurrence (months)	15	16	26
Median followup (months)	25	47	34
Recurrence			
Single tumor	80%	80%	46%
Multiple tumors	20%	20%	54%
Local	41%	22%	13%
Distant	44%	58%	34%
Both	15%	20%	54%
Treatment			
Surgery alone	56%	35%	42%
Surgery + C a/o RT∗	44%	65%	58%
Survival factors			
Time to 1st recurrence	Yes	Yes	Yes
Complete resection	Yes	Yes	Yes
Single tumor	NR	Yes	Yes
FIGO (2009)	NR	NR	Yes
Response to C a/o RT∗	No	Yes	No
Overall survival			
2 years	70%	55%	57%
5 years	28%	25%	24%
10 years	NR	20%	12%

*C: chemotherapy; RT: radiation therapy; NR: not reported.
